# Solid predominant histologic subtype and early recurrence predict poor postrecurrence survival in patients with stage I lung adenocarcinoma

**DOI:** 10.18632/oncotarget.12540

**Published:** 2016-10-09

**Authors:** Jizhuang Luo, Rui Wang, Baohui Han, Jie Zhang, Heng Zhao, Wentao Fang, Qingquan Luo, Jun Yang, Yunhai Yang, Lei Zhu, Tianxiang Chen, Xinghua Cheng, Qingyuan Huang, Yiyang Wang, Jiajie Zheng, Haiquan Chen

**Affiliations:** ^1^ Department of Thoracic Surgery, Shanghai Chest Hospital, Shanghai Jiao Tong University, Shanghai, China; ^2^ Department of Pulmonary Medicine, Shanghai Chest Hospital, Shanghai Jiao Tong University, Shanghai, China; ^3^ Department of Pathology, Shanghai Chest Hospital, Shanghai Jiao Tong University, Shanghai, China; ^4^ Department of Shanghai Lung Tumor Clinic Center, Shanghai Chest Hospital, Shanghai Jiao Tong University, Shanghai, China; ^5^ Department of Thoracic Surgery, Fudan University Shanghai Cancer Center, Shanghai, China; ^6^ Institutes of Biomedical Sciences, Fudan University, Shanghai, China; ^7^ Department of Oncology, Shanghai Medical College, Fudan University, Shanghai, China

**Keywords:** invasive lung adenocarcinoma, non-small cell lung cancer, postrecurrence survival, solid, stage I

## Abstract

**Introduction:**

This study investigated the correlation between histologic predominant pattern and postrecurrence survival (PRS), and identified the clinicopathologic factors influencing PRS in patients with completely resected stage I lung adenocarcinoma.

**Methods:**

A total of 136 stage I lung adenocarcinoma patients who experienced tumor recurrence after completely resection were included in this study. To analysis the association between histologic predominant pattern and PRS, invasive adenocarcinomas with mixed histologic components were divided into 2 groups: solid and nonsolid group (including lepidic, acinar, papillary, micropapillary) based on the histologic predominant pattern. PRS was analyzed to identify the prognostic predictors using the Kaplan-Meier approach and multivariable Cox models.

**Results:**

For all stage I invasive adenocarcinoma patients, the majority of postsurgical recurrences occurred within 2 years. Patients with solid predominant histological pattern were associated with unfavorable PRS (HR, 2.40; 95%CI 1.13-5.08, p=.022). There was a significant difference for poor PRS for patients who diagnosed tumor recurrence shorter than 12 months after surgery (HR, 2.34; 95%CI 1.12-4.90, p=.024). Extrathoracic metastasis was associated with poor media PRS in univariable analysis (p =.011), however, there was no significant PRS difference in multivariable analysis (HR, 1.56; 95%CI 0.65-3.73, p=.322) compared with intrathoracic metastasis.

**Conclusions:**

Solid predominant histologic subtype and recurrence free interval less than 12 months predict worse PRS in patients with stage I lung adenocarcinoma.

## INTRODUCTION

Lung cancer continues to be one of the most common causes of cancer-related death worldwide [1]. Adenocarcinoma is the most common histologic subtype, more than 80% to 90% of which demonstrated heterogeneous histologic patterns [2]. Despite the current standard completely surgical resection for patients with stage I non-small cell lung cancer (NSCLC), 30%-40% of these patients still will relapse [3]. The risk of recurrence peaked within the first 2 years after surgery [4, 5], and the majority of postoperative recurrence were detected in an asymptomatic condition during the regular follow-up period [6]. Previous reports demonstrated that gender, initial recurrence site, postrecurrence therapy and tumor cell differentiation were the prognostic factors of postrecurrence survival (PRS) [7]. However, the PRS for early stage lung adenocarcinoma patients still have not been thoroughly studied.

Since the new classification system for lung adenocarcinoma proposed by International Association for the Study of Lung Cancer (IASLC), American Thoracic Society (ATS) and European Respiratory Society (ERS) in 2011 [8], solid predominant histological subtype gradually proved to be associated with poorer recurrence free survival ( PRS) and overall survival (OS) [2, 9–12]. To date, few studies have focused on the correlation between histologic subtypes of lung cancer and the postrecurrence survival. Shimada et al [13] indicated that nonadenocarcinoma had a significant favorable PRS, while another large cohort study demonstrated that solid predominant histologic pattern was associated with shorter postrecurrence survival [14]. However, more studies are required to validate the conclusion derived from homogeneous research cohort. In this study, we examined the correlation between histologic predominant pattern and PRS, and identified other clinicopathologic factors influencing PRS. Our findings help to guide the postoperative management of patients with completely resected stage I invasive lung adenocarcinoma.

## RESULTS

The media follow-up time for all stage I lung invasive adenocarcinoma patients was 39.93 months (range, 0.3 to 84.4 months). For the 136 patients who experienced recurrence, there were 70 (51%) males, and 66 (49%) females. The number of patients in age less than or equal to 65 and greater than 65 years old were 87 (64%) and 49 (36%). 10 (7%) patients with tumor size greater than 4cm, 64 (47%) patients with visceral pleural invasion, and 19 (14%) patients with lymphovascular invasion. Patients in stage T1a, T1b and T2a were 31 (23%), 35 (26%) and 70 (51%). 70 (51%) patients had received adjuvant chemotherapy and the majority patients (86%) had received lobectomy. According to the 2011 IASLC/ATS/ERS lung adenocarcinoma classification, 136 patients were divided into 2 subgroups. 20(15%) patients were in solid predominant group and 116(80%) patients were in nonsolid group. For the latter group, 2 showed lepidic, 58 showed acinar, 55 showed papillary, 1 showed micropapillary predominant pattern. Table 1 compares the demographic features of 20 solid predominant patients to 116 nonsolid predominant patients.

**Table 1:** Clinicopathologic variables in 136 patients with stage I lung adenocarcinoma

Characteristics	Total No. (%)	Solid No. (%)	Non-solid No. (%)	p
Total of patients	136	20(15)	116(85)	
Age				.321
≤65	87(64)	15(75)	72(62)	
>65	49(36)	5(25)	44(38)	
Gender				.091
male	70(51)	14(70)	56(48)	
female	66(49)	6(30)	60(52)	
Tumor size(cm)				.165
≤4	126(93)	17(85)	109(94)	
>4	10(7)	3(15)	7(6)	
Visceral pleural invasion				<.999
Yes	64(47)	9(45)	55(47)	
No	72(53)	11(55)	61(53)	
Lymphovascular invasion				.482
Yes	19(14)	4(20)	15(13)	
No	117(86)	16(80)	101(87)	
Type of Surgery				.777
Lobectomy	117(86)	18(90)	99(85)	
Wedge resection	17(13)	2(10)	15(13)	
Others	2(1)	0	2(2)	
Adjuvant chemotherapy				.335
Yes	70(51)	8(40)	62(53)	
No	66(49)	12(60)	54(47)	
T stage				.880
1a	31(23)	4(20)	27(23)	
1b	35(26)	6(30)	29(25)	
2a	70(51)	10(50)	60(52)	
TNM stage				<.999
IA	66(49)	10(50)	56(48)	
IB	70(51)	10(50)	60(52)	

### Pattern of initial recurrence

Of the 136 patients who experienced tumor relapse, 38(28%) had local recurrence, 98 (72%) had distant recurrence. As to the recurrence site, 76(56%) had intrathoracic recurrence, 60 (44%) had extrathoracic recurrence. 66% (n = 90) of the recurrence were found at a single site, while 34% (n = 46) of the recurrence were diagnosed at multiple site. There were no significant different between solid and nonsolid group for the recurrence pattern (Table 2).

**Table 2:** Correlation between histologic predominant subtypes and initial recurrence site

Variable	Total No. (%)	Solid No. (%)	Non-solid No. (%)	p
Total No. of patients	136	20(15)	116(85)	
Recurrence pattern				.189
Local	38(28)	3(15)	35(30)	
Distant	98(72)	17(85)	81(70)	
Recurrence pattern				.147
Intrathoracic	76(56)	8(40)	68(59)	
Extrathoracic	60(44)	12(60)	48(41)	
Recurrence pattern				.308
Single site	90(66)	11(55)	79(68)	
Multiple site	46(34)	9(45)	37(32)	

### Postrecurrence survival analysis

**Figure 1:**
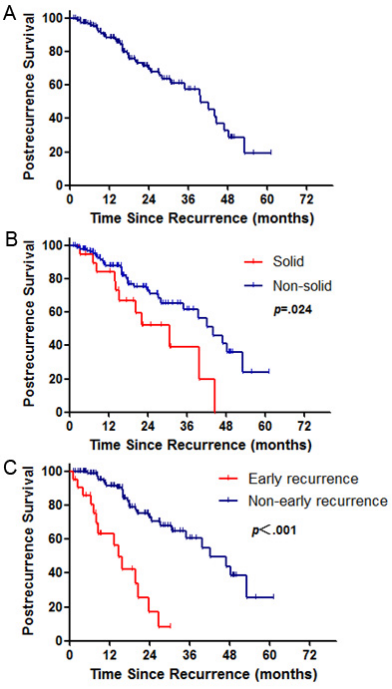
Survival cures for postrecurrence survival **A.** Survival cures according to histological predominant pattern **B.** and recurrence-free interval **C.** for postrecurrence-free survival in stage I lung adenocarcinoma patients. p values from log-rank test.

For the 136 patients who experienced recurrence, 41(30%) died (37 as a result of cancer-related disease). The median PRS was 15.97 months. There was a significant worse PRS (p = .024) for solid predominant pattern compared with nonsolid predominant pattern (Figure 1B). In order to identify weather the PRS was related to the period of recurrence free survival, we performed the survival analysis on two subgroups with different recurrence-free interval. Statistical significance were seen for poor PRS in early recurrence group (recurrence-free interval ≤12 months; p < 001; Figure 1C).

### Risk factors for postrecurrence survival

In univariable analysis, for primary tumor factors, no significant median PRS difference were seen in age, tumor size, visceral pleural invasion, lymphovascular invasion, TNM stage and adjuvant chemotherapy. Media PRS were significantly shorter in male (p = .022) and solid predominant histological pattern (p = .024). For postrecurrence factors, recurrence free interval less than 12 months (p = .001) and extrathoracic recurrence (p = .011) predict poorer median PRS (Table 3).

**Table 3:** Univariable analysis of postrecurrence survival

Characteristic	No. (%)	Median PRS (95%CI), Months	p
Total No. of patients	136		
Primary tumor factor			
Age			.671
≤65	87 (64)	42.2 (8.1-55.8)	
>65	49 (36)	39.6 (7.3-42.6)	
Gender			.022
male	70 (51)	28.5 (4.0-55.8)	
female	66 (49)	49.9 (9.4-60.9)	
Tumor size			.995
≤4cm	126 (93)	39.7 (7.1-60.9)	
>4cm	10 (7)	NA	
Visceral pleural invasion			.806
Absence	72 (53)	39.6 (5.4-55.8)	
Present	64 (47)	42.2 (8.1-60.9)	
Lymphovascular invasion			.626
Absence	117 (86)	39.7 (7.1-60.9)	
Present	19 (14)	48.4 (9.5-31.0)	
TNM stage			.696
IA	66 (49)	39.6 (5.4-55.8)	
IB	70 (51)	42.2 (8.1-60.9)	
Pathologic subtypes			.024
Solid	20 (15)	30.8 (3.1-39.7)	
Nonsolid	116 (85)	43.9 (8.6-60.9)	
Adjuvant chemotherapy			.897
No	66 (49)	39.7 (6.1-49.9)	
Yes	70 (51)	42.2 (7.3-60.9)	
Postrecurrence factors			
Recurrence-free interval, months			.001
≤12	22 (16)	28.0 (3.1-43.9)	
>12	114 (84)	42.2 (10.0-60.9)	
Recurrence pattern			.161
Local	38 (30)	48.4 (3.1-55.8)	
Distant	98 (70)	39.6 (8.1-60.9)	
Recurrence pattern			.011
Intrathoracic	76 (56)	48.4 (7.1-60.9)	
Extrathoracic	60 (44)	39.6 (8.3-44.6)	
NA= not applicable			

Multivariable Cox models stratified by trial were performed to identify risk factors for PRS. For all recurrence patients, multivariable survival analysis adjusting gender, age, tumor size, visceral pleural invasion, lymphovascular invasion, adjuvant chemotherapy, histological pattern and TNM stage, showing that solid predominant pattern was an independent risk factor for poor PRS (HR, 2.40; 95%CI, 1.13-5.08; p = .022) among primary tumor factors. Early recurrence was the only independent risk factor for worse PRS (HR, 2.34; 95%CI, 1.12-4.90; p = .024) among postrecurrence factors (Table 4).

**Table 4:** Multivariable analysis in predicting postrecurrence survival

Factor	Multivariable		
Primary tumor factors	HR	95%CI	p
Age (>65 vs. ≤65)	1.26	0.60-2.66	.540
Gender (male vs. female)	1.01	0.52-2.00	.967
Tumor size (>4cm vs. ≤4cm)	1.14	0.33-3.93	.839
Visceral pleural invasion ( Yes vs. No)	1.49	0.16-13.60	.727
Lymphovescular invasion ( Yes vs. No)	0.63	0.21-1.85	.396
TNM stage (IA vs. IB)	0.64	0.06-6.33	.699
Adjuvant chemotherapy (Yes vs. No)	1.18	0.60-2.31	.640
Pathologic subtype (solid vs. nonsolid)	2.40	1.13-5.08	.022
Postrecurrence factors			
Recurrence-free interval (≤12 vs.>12 months)	2.34	1.12-4.90	.024
Recurrence pattern			
Distant vs. local	1.17	0.46-2.97	.746
Extrathoracic vs. intrathoracic	1.56	0.65-3.73	.322

## DISCUSSION

The retrospective study showed that solid predominant histological pattern was an independent primary tumor predictor for unfavorable postrecurrence survival for stage I lung adenocarcinoma patients. The majority of postsurgical recurrences occurred within 2 years. There was a significant difference for poor PRS for patients who diagnosed recurrence shorter than 12 months after surgery. Extrathoracicmetastasis was associated with poor media PRS in univariable analysis, however, there was no significant PRS difference in multivariable analysis compared with intrathoracic metastasis.

Generally, in the IASLC/ATS/ERS classification system, solid predominant pattern have not obviously morphologically recognizable differentiation features and been recognized as poorly differentiated carcinomas [2, 15]. A numerous studies indicated that invasive adenocarcinoma with solid component was associated with poorer recurrence free interval [16–19]. Few researches used the new adenocarcinoma classification in predicting postrecurrence survival. Consistent with our findings, Ujiie and colleagues [14] indictaed that on multivariable analysis, solid predominant histologic pattern independently associated with worse PRS, with the 8.7 median postrecurrence survival months. Poorly differentiation, higher risk of visceral pleural invasion and occult lymph node metastasis may account for the poor prognosis.

Recurrence free interval less than 12 months was the only independent postrecurrence predictors of unfavorable prognosis in our study. Several previous reports [20–22] demonstrated that recurrence free interval in stage I NSCLC after completed tumor resection as a significant prognostic factor. Walsh and co-workers [23] pointed that recurrence-free interval was a direct measure of patient's tumor biology. Hung and colleagues [5] indicated that recurrence free interval less than 16 months still had a significant negative effect on PRS. Our results found that compared with nonsolid group, solid predominant histologic pattern had a significant shorter recurrence free interval. We next analyzed the association between recurrence pattern and recurrence free interval. There were no statistical significant difference for recurrence pattern (local/distant or intrathoracic/extrathoracic) and number of recurrence sites between two different recurrence free interval groups. The worse PRS for early recurrence patients may be related to rapid progression of the disease.

For others postrecurrence factors in predicting PRS, several published reports [4, 6] indicated that distant, extrathoracic metastasis and symptoms at the initial recurrence were predictors for poor PRS. Sugimura and co-workers [22] demonstrated that initial recurrence confined to lungs was correlation to better postrecurrence survival. In our study, the media PRS were 39.6 months in patients harbored extrathoracic metastasis, which significantly shorter than patients only detected intrathoracic recurrence. Advanced large cohorts are warranted to further confirm the conclusion.

There were several studies focused on the effect of postrecurrence therapy (PRT) on PRS among patients with resected stage I NSCLC. In Shimada's [13] cohort, the majority patients who experienced recurrence received initial chemotherapy or second-line chemotherapy. Multivariate analysis indicated that PRT had a strong impact on PRS. In Hung's cohort [5], no significant difference was seen in PRS between patients received re-operation and those who had chemotherapy and/or radiotherapy, but the conclusion needed further investigation. EGFR mutations were less likely to detect in solid predominant patients, which predicts primary resistance to tyrosine kinase inhibitors (TKIs) [24, 25]. This may another reason for the poor PRS among patients with solid predominant pattern. The underlying mechanism may validate by immunology, molecular biology and circulating tumor cells detect methods.

There were some limitations in the current study. Firstly, the retrospective study had an insufficient patient cohort, which only included 136 patients and selection bias is inevitable. Secondly, we used a homogeneous cohort, so the distribution of clinicopathologic characteristics might be different in other countries, patients selective bias was inevitable. The frequencies of the histologic predominant subtype of lung adenocarcinoma vary in previous reports [26, 27].The reason may causes by the different diagnostic experiences among pathologists, which may affect the conclusions. Thirdly, patients might receive surgery, chemotherapy, radiotherapy or immunotherapy after been diagnosed tumor recurrence, however, in this study, we didn't analysis patients’ postrecurrence therapy, which may affect the postrecurrence survival.

In summary, solid predominant histological subtype has more aggressive behavior and is associated with unfavorable PRS for stage I lung adenocarcinoma patients. Patients who diagnosed tumor recurrence with 12 months after surgery have a significantly worse postrecurrence survival.

## MATERIALS AND METHODS

### Patients cohort

Institutional review board approval was obtained for this retrospective study from Shanghai chest hospital. Patients who underwent surgical resection for stage I lung invasive adenocarcinoma at Shanghai Chest Hospital between January 2009 and March 2015 were retrospectively reviewed. Patients who had multiple nodules, history of malignancies, adenocarcinomas in situ (AIS), minimally invasive adenocarcinoma (MIA), or had received neoadjuvant chemotherapies were excluded from this study. A total of 3536 patients with stage I lung adenocarcinoma were identified. Among them, 136 patients who experienced recurrence with complete medical records were included in our cohort.

### Clinicopathological evaluation

For the 136 patients, two pathologists independently reviewed all hematoxylin and eosin (HE) stained slides of the surgically resected specimens. The criteria of the IASLC/ATS/ERS lung adenocarcinoma classification were used for histologic classification. [8] Invasive adenocarcinomas were divided into 5 subtypes: lepidic (LEP), acinar (ACN), papillary (PAP), micropapillary (MIP) and solid (SOL) predominant subgroup based on the greatest percentage of tumor histology presented in the slides, the percentage of each histologic pattern in 5% increments was recorded. For few of patients with LEP or MIP predominant pattern in our cohort, we divided the predominant pattern group into nonsolid (LEP, ACN, PAP and MIP) or solid group.

The TNM Staging was based on the 7th edition of the American Committee on Cancer (AJCC) cancer staging manual [28]. The clinicopathologic features including age, gender, tumor size, visceral pleural invasion, lymphovascular invasion and recurrence pattern were collected from medical records.

### Surveillance protocol

For all patients, preoperative examination including head, chest and upper abdomen CT scans, pulmonary function testing to exclude multiple nodules and distant metastasis. However, not all patients received postoperative follow-up in our hospital. For patients who were follow-up regularly in our out-patients center, the postoperative surveillance was performed as in our previous publication [29]: physical examination, blood tests, chest CT, neck and upper abdominal ultrasound examination were performed in every 3 months for the first year after tumor resection and at 6-month intervals thereafter. Whole-body bone scanning and magnetic resonance imaging (MRI) of the brain were performed annually. Additional imaging studies were performed if patients had any symptoms or signs of recurrence occurred regardless of the follow-up schedule. For patients who did not follow-up in out hospital, a telephone follow up were conducted to record the survival status as well as the results of previous examination performed in other hospitals. In order to accurately study the correlation between recurrence pattern and predominant histology subtypes, we excluded the patients who were obtained survival status from telephone follow-up for the incomplete medical records.

Recurrence-free interval as primary end point was defined as time from surgery to the date of first event (recurrence or metastasis). Postrecurrence survival as another end point was defined as time from recurrence to the date of death resulting from any cause. Local recurrence was defined as any new lesion in contiguous anatomic sites, including the ipsilateral hemithorax and mediastinum. Distant recurrence was defined as tumor recurrence in the contralateral lung, contralateral mediastinum or outside the hemithorax and mediastinum with or without local recurrence. Intrathoracic recurrence was defined as tumor recurrence occurred in the ipsilateral or contralateral hemithorax and mediastinum. Extrathoracic recurrence was defined as tumor recurrence out of the bilateral hemithorax with or without intrathoracic recurrence. Initial recurrence was defined as the site of the initial discovery of tumor recurrence. A combination recurrence was defined as the both local and distant or both intrathoracic and extrathoracic metastasis discovered within 30 days.

### Statistical methods

The χ2 test was used to compare between groups with respect to categorical and continuous variables. Cochran-Mantel-Haenszel test was used to estimate PRS. The log-rank test was used to compare the differences in PRS between histologic groups for univariable analysis. Multivariable Cox models stratified by trial and adjusted for gender, age, tumor size, visceral pleural invasion, lymphovascular invasion, histologic subtypes, adjuvant chemotherapy and TNM stage were used to measure the prognostic value in postrecurrence survival. The value of statistical significance was set to .05 (pooled analysis). Statistical analyses were performed using SPSS software (version 19; SAS Institute, Cary, NC) and GraphPad (Prism 5).
